# Diversity and phylogenetic analysis of endosymbiotic bacteria of the date palm root borer *Oryctes agamemnon* (Coleoptera: Scarabaeidae)

**DOI:** 10.1186/s12866-015-0422-8

**Published:** 2015-04-22

**Authors:** Wael S El-Sayed, Reda A Ibrahim

**Affiliations:** Biology Department, Faculty of Science, Taibah University, Almadinah Almunawarah, 344 Saudi Arabia; Microbiology Department, Faculty of Science, Ain Shams University, Cairo, 11566 Egypt; Department of Economic Entomology, Kafrelsheikh University, Kafr El-Sheikh, 33516 Egypt

**Keywords:** *Oryctes agamemnon*, Endosymbionts, 16S rRNA gene, DGGE

## Abstract

**Background:**

The date palm root borer *Oryctes agamemnon* (Coleoptera: Scarabaeidae) is one of the major pests of palms. In Saudi Arabia, both larvae and adults of *Oryctes* are particularly troublesome, especially during the establishment of young date palm orchards. Endosymbiotic bacteria are known to have a key role in food digestion and insecticide resistance mechanisms, and therefore are essential to their host insect. Identification of these bacteria in their insect host can lead to development of new insect pest control strategies.

**Results:**

Metagenomic DNA from larval midgut of the date palm root borer, *O. agamemnon*, was analyzed for endosymbiotic bacterial communities using denatured gradient gel electrophoresis (DGGE) utilizing 16S rRNA genes. The DGGE fingerprints with metagenomic DNA showed predominance of eleven major operational taxonomic units (OTUs) identified as members of *Photobacterium*, *Vibrio*, *Allomonas*, *Shewanella*, *Cellulomonas*, and *Citrobacter*, as well as uncultured bacteria, including some uncultured *Vibrio* members. DGGE profiles also showed shifts in the dominant bacterial populations of the original soil compared with those that existed in the larval midguts. The endosymbiotic bacterial community was dominated by members of the family *Vibrionaceae* (54.5%), followed by uncultured bacteria (18.2%), *Enterobacteriaceae* (9.1%), *Shewanellaceae* (9.1%), and *Cellulomonadaceae* (9.1%). Phylogenetic studies confirmed the affiliation of the dominant OTUs into specified families revealed by clustering of each phylotype to its corresponding clade. Relative frequency of each phylotype in larval midguts revealed predominance of *Vibrio furnisii* and *Vibrio navarrensis*, followed by uncultured bacterial spp., then *Cellulomonas hominis*, *Shewanella algae*, and *Citrobacter freundii*.

**Conclusion:**

Analysis of metagenomic DNA for endosymbiotic bacterial communities from the midgut of *Oryctes* larvae showed strong selection of specific bacterial populations that may have a key role in digestion, as well as other benefits to the larvae of *O. agamemnon*. Determination of the distinct endosymbiotic community structure and its possible biological functions within the insect could provide us with basic information for future pest control research.

## Background

Several insect pests attack date palm (*Phoenix dactylifera* L.) orchards, causing serious damage and economic losses. In many Arabian countries, three species of rhinoceros beetles, *Oryctes* (Coleoptera: Scarabaeidae), *O. elegans, O. agamemnon* and *O. rhinoceros*, are known to infest date palm orchards [1]. The most widespread is *O. agamemnon*, which is a root borer in its larval stage and a frond borer in the adult stage. The other two species, *O. rhinoceros* and *O. elegans*, are fruit stalk borers and can also act as root borers [2]. *Oryctes* spp. have a wide host range, attacking and causing serious damage and crop loss on many hosts, including date palm, coconut palm, betel nut, sago palm and oil palm [3]. Recently, *Oryctes* spp. have emerged as major pests of different date palm cultivars. In Saudi Arabia, both larvae and adults of *Oryctes* are particularly troublesome, especially during the establishment of young date palm orchards. The development time of the larval stage is long and may extend for several years in some species. The larvae feed on roots and rotten wood whereas the adults feed on nectar, plant sap and fruit [4-7].

Certain mutualists may influence host plant range and enable insect pests to modify plant physiology for their own benefit. There is increasing evidence for the role of microbial mutualistic symbioses in insect–plant interactions [8]. The horizontal transmission of mutualists among their host insects can be achieved through a route involving its host plant. Where this transmission occurs, the insect mutualist might either become a plant pathogen and damage the plant or change the way the plant interacts with its natural enemies and host competitors [9].

Insect intestinal tracts harbor rich communities of nonpathogenic microorganisms [10]. A single gut can harbor 10^5^–10^9^ prokaryotic cells [11] that have been affiliated to twenty-six phyla, at least for the insects studied to date. It is increasingly evident that insect microbiota are essential for normal growth and development [12]. It has been shown that about 65% of insects possess symbiotic bacteria. *Wolbachia* spp. is the most commonly reported genus [13-15]. The symbiotic relationship between bacteria and insects varies from being mutualistic and commensal to pathogenic [16,17]. Based on their role, intracellular symbionts in insects are classified as primary or secondary endosymbionts. Primary (obligate) symbionts are essential for the insect due to their role in nutrient supplementation, whereas secondary symbionts have a useful but not essential role for insect survival [18,19]. Insect endosymbionts are detected in specific organs referred to as bacteriomes or mycetomes, usually resulting in a strict vertical transmission from mother to offspring.

Understanding relationships between endosymbiotic bacteria and their insect hosts is not only relevant from an evolutionary view, but can also lead to the identification of new targets for insect pest control [20]. Since many of the relevant endosymbionts cannot be cultured, their functional characterization and/or identification has been difficult. Certain symbionts have been developed as biological control agents and were found to be effective against Chagas disease vectored by *Rhodnius prolixus*. In this example, the endosymbiotic organism, *Rhodococcus rhodnii* was genetically transformed to express an anti-trypanosomal output in the insect gut [21].

The date palm root borers of the genus *Oryctes* are regarded as devastating and invasive pests in a wide variety of palms worldwide. Little is known about the presence of endosymbionts in the genus *Oryctes*. Exploring bacteria-insect associations in this regard would be useful for potential insect pest control. For example, if obligate endosymbionts exist in *Oryctes*, then eliminating them using baits could be a potential control strategy. Investigation of endosymbiosis in this genus may help to understand the host-symbiont interactions and the evolution of different reproductive strategies in these beetles, and ultimately provide a future basis for development of novel pest management strategies. Therefore, the objective of this study was to analyze the diversity of the larval midgut microbiota of the date palm root borer, *O. agamemnon*.

## Results and discussion

### Endosymbionts of *Oryctes agamemnon* larvae

**Figure 1:**
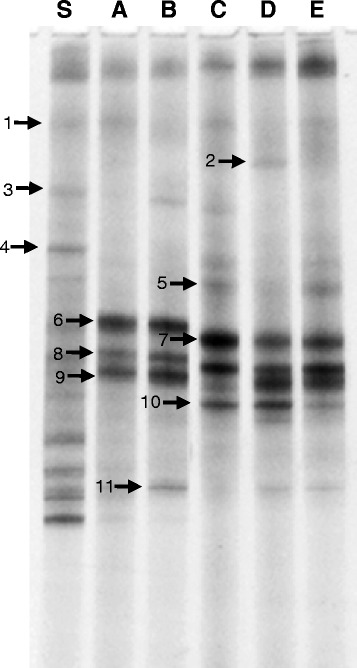
DGGE fingerprints of endosymbiotic bacterial communities from five *O. agamemnon* larvae midguts **(A, B, C, D,** and **E)** compared with native bacterial populations in inhabited soil **(S)**.

Microbial diversity is defined as the number of elements indicated by species or genes within a system [22]. Most of the microbial world within a system remains unexplored due to the existence of many uncultured bacteria species. Molecular-based approaches are useful for determining diversity of various bacterial populations [23-25]. Several molecular methods based on DNA analyses using polymerase chain reaction (PCR) followed by an analysis of the diversity of PCR products are available [26-28]. Polymerase chain reaction denatured gradient gel electrophoresis (PCR-DGGE) [26] in particular, has been regarded as a powerful genetic fingerprinting technique for evaluation of bacterial community structures in different environmental niches. PCR-DGGE analysis utilizing 16S rRNA genes usually yield patterns that reflect the composition of the dominant microorganisms, including the uncultured members [24]. DGGE has been widely used to investigate several bacterial patterns in soil [29], marine habitats [30], rhizosphere [31], grasslands [32], manure and fertilizers [33], and sites polluted with anthropogenic chemicals [34]. Bacterial diversity and community structure of insect endosymbiotic bacteria have not been investigated by DGGE previously. Therefore, we used DGGE in this study to investigate bacterial populations in the midgut of *O. agamemnon* larvae. The DGGE patterns obtained with total community DNA from larval midguts showed predominance of eleven major OTUs (Figure 1). DGGE profile of metagenomes belonging to five larval midguts showed the same pattern, confirming a stable and intact endosymbiotic bacterial community structure. DGGE was also used to investigate the distribution pattern of soil bacteria in larva-infested soil. DGGE fingerprinting showed changes in the dominant bacterial populations of the original soil compared with those that existed in the midguts. This shift could be attributed to the strong selection of specific bacterial populations that may have a key role in insect nutrition. The consistency of such midgut endosymbionts suggests the presence of *O. agamemnon*-specific microbiota. Andert et al. [35] addressed the question whether or not the hindgut of the two closely related scarabs *Pachnoda ephippiata* and *Pachnoda marginata*, harbors a specific bacterial microbiota. Terminal restriction fragment length polymorphism (T-RFLP) analysis showed that in both species, the hindgut bacterial community strongly differs from that in the midgut, food soil, and fecal pellets. It was concluded that high intra- and interspecific similarities between the T-RFLP profiles of different larvae indicate the presence of a hindgut-specific microbiota.

### Endosymbiotic bacterial community structure

Endosymbiotic bacterial community structure for insects in general based on culture dependent methods will remain uncertain. Culture-independent methods allow a deeper understanding of the composition of microbial communities in different ecosystems [23]. In this study we examined the endosymbiotic bacterial community structure of date palm root borer larvae with DGGE for rapid comparison of data from many communities and specific phylogenetic information derived from excised bands [27]. Sequence analysis of 16S rRNA gene fragments obtained from DGGE revealed the identity of the endosymbionts in the larval midgut of *O. agamemnon*. Based on BLAST best similarity matches, phylotypes frequently detected in fingerprinting analysis have been affiliated to *Photobacterium* sp., *Vibrio* sp., *Allomonas* sp., *Shewanella* sp., *Cellulomonas* sp., and *Citrobacter* sp., as well as some uncultured bacteria, including uncultured *Vibrio* sp. (Table 1). These bacteria might be responsible for palm tissue fermentation in the tunnels where *O. agamemnon* larvae thrive and might have a key role in the insect’s nutrition. Many of the Enterobacteriaceae produce digestive enzymes and therefore have a role in insect nutrition [36].

**Table 1:** **Bacterial species identified in the midgut of** ***O. agamemnon*** **larvae**

**DGGE band**	**Accession no.**	**Closest matches**			**Phylogenetic affiliation**
		**Identity**	**Accession no.**	**Similarity (%)**	
1	LC009469	*Photobacterium ganghwense* FR1311	NR043295	100	Gammaproteobacteria/Vibrionaceae
		*Photobacterium* sp.	AB583193	100	Gammaproteobacteria/Vibrionaceae
		*Photobacterium* sp. RSBAUOCAS0005B	HM641040	100	Gammaproteobacteria/Vibrionaceae
2	LC009470	*Vibrio fluvialis* MBTD-CMFRI-Vf05	KF317830	100	Gammaproteobacteria/Vibrionaceae
		*Vibrio* sp. BTOK10	JQ923505	100	Gammaproteobacteria/Vibrionaceae
		*Vibrio vulnificus* MP-4	AY911393	100	Gammaproteobacteria/Vibrionaceae
3	LC009471	*Photobacterium ganghwense* FR1311	NR043295	100	Gammaproteobacteria/Vibrionaceae
		*Vibrio fortis* H083	KJ577078	100	Gammaproteobacteria/Vibrionaceae
		Uncultured bacterium clone SWH04_PR	JQ480712	98	Gammaproteobacteria/Vibrionaceae
4	LC009472	Uncultured bacterium clone BT12G08	KC208438	99	Bacteria/Environmental sample
		Uncultured bacterium clone SWG11_MS	JQ480736	99	Bacteria/Environmental sample
		Uncultured bacterium clone nbw223h08c1	KF064992	99	Bacteria/Environmental sample
5	LC009473	*Allomonas enterica* JC102, D09-37	FR837603	98	Gammaproteobacteria/Vibrionaceae
		Uncultured *Vibrio* sp. clone D004025F04	GU179548	98	Gammaproteobacteria/Vibrionaceae
		Uncultured bacterium clone LGH02-B-135	HQ916550	98	Gammaproteobacteria/Vibrionaceae
6	LC009474	*Vibrio navarrensis* AM37820	KJ807107	100	Gammaproteobacteria/Vibrionaceae
		*Vibrio navarrensis* 2544-86	KJ807099	100	Gammaproteobacteria/Vibrionaceae
		*Vibrio navarrensis* 1397-6T	KJ807092	100	Gammaproteobacteria/Vibrionaceae
7	LC009475	*Vibrio* sp. U15	HF968434	100	Gammaproteobacteria/Vibrionaceae
		*Vibrio furnisii* (ATCC 35016T)	X74704	100	Gammaproteobacteria/Vibrionaceae
		Uncultured *Vibrio* sp. clone KR-SUC-9-A10	AM183773	99	Gammaproteobacteria/Vibrionaceae
8	LC009476	*Shewanella algae* H5	KM007068	99	Gammaproteobacteria/Shewanellaceae
		*Shewanella haliotis* NIOT-CS16	KJ371072	99	Gammaproteobacteria/Shewanellaceae
		*Shewanella* sp. MPTDBS	KJ796480	99	Gammaproteobacteria/Shewanellaceae
9	LC009477	*Cellulomonas hominis* PuiC5.18	LM994741	99	Actinobacteria/Cellulomonadaceae
		*Cellulosimicrobium cellulans* S17	KJ947163	99	Actinobacteria/ Promicromonosporaceae
		*Cellulomonas aerilata* JCM 16376	AB910521	99	Actinobacteria/Cellulomonadaceae
10	LC009478	*Citrobacter freundii* C09	KM222617	99	Gammaproteobacteria/Enterobacteriaceae
		*Citrobacter youngae* GTC 01314	AB741661	99	Gammaproteobacteria/Enterobacteriaceae
		*Citrobacter murliniae* M-T-MRS_22	JQ795823	99	Gammaproteobacteria/Enterobacteriaceae
11	LC009479	Uncultured bacterium clone SWH04_PR	JQ480712	98	Bacteria/Environmental sample
		Uncultured bacterium clone BT12G08	KC208438	99	Bacteria/Environmental sample
		Uncultured bacterium clone SWG11_MS	JQ480736	99	Bacteria/Environmental sample

Gut bacteria have been reported to exert many useful functions, such as preventing disease, degrading insecticides, and directly or indirectly contributing to food digestion [15]. Food materials may be important in regulating the dynamics of the bacterial community within the insect gut. For example, *S. marcescens* is a facultative anaerobe that aids in consuming oxygen at the periphery of the Formosan termite’s stomach, thereby maintaining a habitable gut for the strict anaerobes that digest cellulose [37]. In addition to aiding digestion, *Citrobacter* detected in our study is believed to have the same role in establishing anaerobic conditions for the succession of *Shewanella* spp. involved in anaerobic fermentation of ingested materials.

Analysis of larval midgut bacterial populations in *O. agamemnon* revealed a predominance of members belonging to the genus *Vibrio*. Dominance of certain bacterial taxa as endosymbionts in some insects has been reported. Using sequence-based bacterial typing, Hirsch et al. [17] identified bacterial endosymbionts in four species of *Otiorhynchus*. More than 90% of all sequence reads belonged to the genus *Rickettsia*. Tagliavia et al. [38] analyzed the gut microbiota of larvae of the red palm weevil. High abundance of Enterobacteriaceae was detected. Fujiwara et al. [39] surveyed symbiotic bacteria from *Bemisia tabaci* species and reported the dominance of *Rickettsia* in all examined whitefly species.

In contrast to our results with larvae, in a study of gut microbiota of adult *Oryctes monoceros* by Desai and Bhamre [40], a completely different microbial population, except for *Citrobacter*, was reported, and included *Dienococcus proteolyticus*, *Micrococcus varians*, *Micrococcus kristinae*, *Micrococcus roseus*, *Micrococcus lylae*, *Citrobacter amalonacticus*, *Corynebacterium xerosis* and *Bacillus fermentas*.

Cellulolytic bacteria are important for digestion of cellulosic materials. In our study, *Cellulomonas* sp. has been detected as a member of the *O. agamemnon* midgut bacterial population indicating its involvement in the digestion process. Huang et al*.* [41] isolated strains of aerobic and facultatively anaerobic cellulolytic bacteria from the gut of *Holotrichia parallela* (Coleoptera: Scarabaeidae) larvae. The cellulolytic bacterial community was dominated by *Proteobacteria*, *Actinobacteria*, *Firmicutes*, and *Bacteroidetes* (1.45%). However, *Cellulomonas* sp. in particular, was not detected among this community.

### Diversity of *Oryctes agamemnon* endosymbionts

The versatility and diversity of insect-bacteria interactions leads to an enormous potential regarding the mechanisms for the modulation and control of insect pests with both medical and agricultural implications [42]. Through TFLP analyses of bacterial rRNA extracted from the guts of *Harpalus pensylvanicus* and *Anisodactylus sanctaecrucis* (Coleoptera: Carabidae), Lundgren et al. [8] revealed that gut-associated bacterial communities were of low diversity. The bacterial community in these beetles comprised *Serratia* sp., *Burkholderia fungorum*, *H. alvei*, *Phenylbacterium* sp., Caedibacter sp., Spiroplasma sp., Enterobacter strain B-14, and *Weissella viridescens*. Some of these organisms, but not all have been previously associated with insects. However, none of them has been detected in *O. agamemnon*, suggesting that their larvae have a unique bacterial community. In comparison to previously reported insect microbiota, our study revealed low diversity and a highly unique pattern for *O. agamemnon* microbiota.

**Figure 2:**
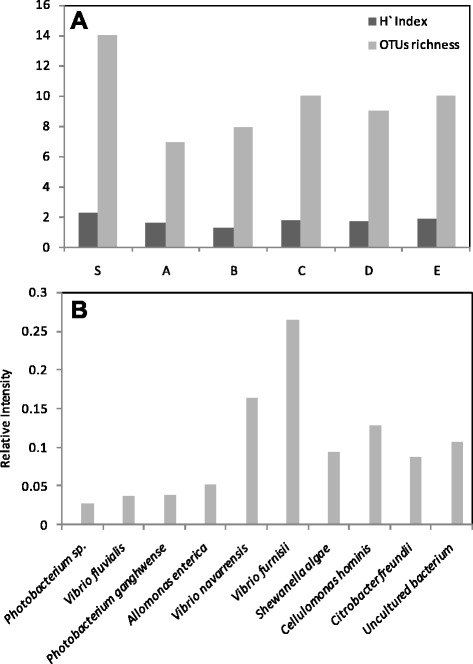
Numerical analysis for the diversity of the *O. agamemnon* endosymbionts. **(A)**, DGGE OTUs richness and Shannon diversity index (*H’*) determined from DGGE fingerprints of endosymbiotic bacterial communities of larvae midguts (A, B, C, D, and E) compared with soil bacteria (S). **(B)**, Relative frequency of each phylotype in larvae midguts.

The midgut bacterial populations of *O. agamemnon* larvae were taxonomically restricted to two major groups, with 80% of the natural bacterial microbiota composed of only three bacterial families within Gammaproteobacteria. The dominant bacterial taxa are members of Vibrionaceae (54.5%), Enterobacteriaceae (9.1%), and Shewanellaceae (9.1%). In addition to Gammaproteobacteria, one family belonging to Actinobacteria was detected (Cellulomonadaceae (9.1%)). Host diet plays a major role in shaping the insect bacterial microbiota. Chandler et al*.* [43] found that Drosophilid flies have taxonomically restricted bacterial communities, with 85% of the natural bacterial microbiome composed of only a few bacterial families (Enterobacteriaceae, Lactobacillales and Acetobacteraceae). 18.2% from the total bacterial population was detected as uncultured bacterial members. Several indices, including species richness and evenness, are used to describe the structural diversity of a community [44]. (Figure 2A) shows the diversity and richness of bacterial species of *O. agamemnon* larvae compared with those of the soil where the larvae live. The reduction in diversity and richness of bacterial species of larvae compared with soil was attributed to the selection of specific bacterial populations that may have a key role in food digestion for the benefit to the larvae. (Figure 2B) shows the relative frequency of each bacterial species and the predominance of *Vibrio* spp. among the endosymbiotic bacterial population.

### Phylogenetic analysis

The 16S rRNA genes are used for phylogenetic affiliation of Eubacteria and Archaea. Partial sequences of 16S rRNA gene of bacterial microbiota from the larval midgut of *O. agamemnon* have been analyzed. Sequences were compared with their closest matches with BLAST search tool to obtain the nearest phylogenetic neighbors. About 72.7% of the bacterial community was assigned to Gammaproteobacteria. The remainder of the bacterial community was assigned to Actinobacteria (9.1%) and uncultured bacterial members (18.2%). Bacteria belonging to Gammaproteobacteria were classified as members of three families; *Vibrionaceae*, *Enterobacteriaceae*, and *Shewanellaceae*, with predominance of the former. Actinobacteria comprised only one family, *Cellulomonadaceae* enclosing *Cellulomonas* sp.

Tagliavia et al. [38] analyzed the gut microbiota of larvae of the red palm weevil. They assigned 98% of the total population to only three phyla: Proteobacteria, Bacteroidetes, and Firmicutes, and three main families (Enterobacteriaceae, Porphyromonadaceae and Streptococcaceae). Bacterial members have been identified as *Dysgonomonas*, *Lactococcus*, *Salmonella*, *Enterobacter*, *Budvicia*, *Entomoplasma*, *Bacteroides* and *Comamonas*. The major phylogenetic microbiota of the hindgut of *P. ephippiata* were identified through a 16S rRNA gene clone library and revealed that Clostridia, Betaproteobacteria, and Bacteroidetes, followed by Bacillales and Deltaproteobacteria, were dominant.

**Figure 3:**
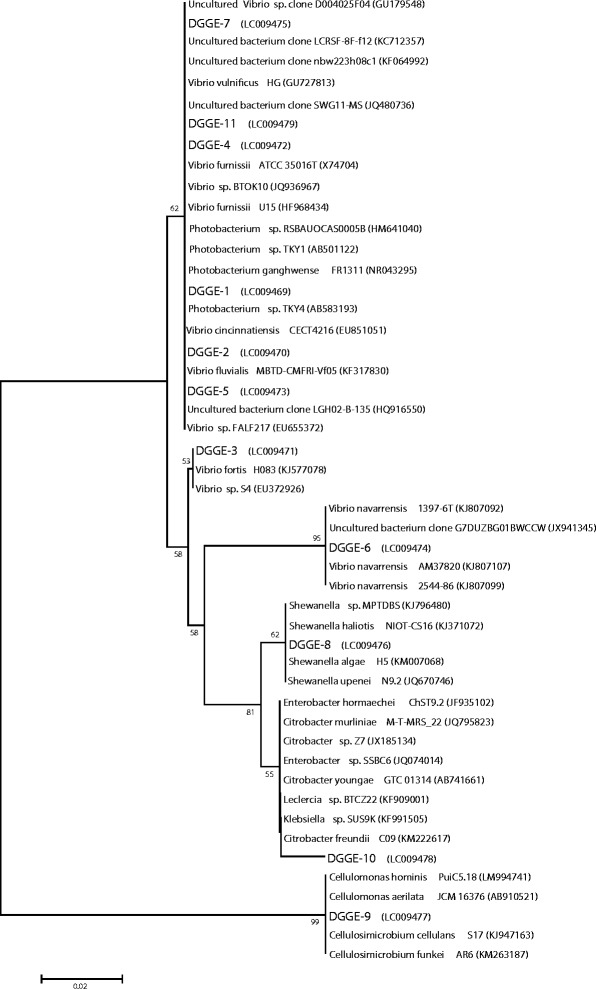
Phylogenetic tree based on the 16S rRNA gene sequence of the *Oryctes agamemnon* endosymbiont in relation to closest matches from NCBI GenBank database with corresponding accession numbers given in parentheses. The tree was constructed by the neighbor-joining method using Kimura’s correction for multiple substitutions. Percent bootstrap values (1000 resamplings) of their level of support are shown at the individual nodes. The scale bar represents 0.02 substitutions per nucleotide position. The data sets supporting the results concerning phylogenetic analysis are available in the Dryad Digital Repository: http://dx.doi.org/10.5061/dryad.59h51.

In this research, phylogenetic studies confirmed the affiliation of dominant OTUs from *O. agamemnon* midgut to members of four distinct families, *Vibrionaceae*, *Shewanellaceae*, *Enterobacteriaceae*, and *Cellulomonadaceae*, revealed by clustering of each individual member to its corresponding group. (Figure 3) represents the phylogenetic tree based on 16S rRNA sequences analysis and showing the relationship between selected dominant phylotypes (OTUs) and representative species, along with other related genera. According to phylogenetic analysis, six phylotypes have been assigned to the family Vibrionaceae including, DGGE-OTU 1, 2, 4, 5, 7, and 11. DGGE-OTU 6 was identified as *Vibrio* member and clustered at a separate phylogenetic branch with *Vibrio navarrensis* indicating its close relation to that species in particular; and finally, DGGE-OTU 3 was specifically clustered with *Vibrio fortis* H083 and *Vibrio* sp. S4. Family Shewanellaceae was found to contain only one species, DGGE-OTU 8, with 99% sequence similarity to *Shewanella* sp. and phylogenetically clustered with *Shewanella* spp. branch. The phylotype DGGE-OTU 9 was affiliated to the Actinobacteria and assigned to *Cellulomonas* sp. (99%) or *Cellulosimicrobium cellulans* S17 (99%). Enterobacteriaceae group was only represented by one phylotype, DGGE-OTU 10, that has been affiliated to *Citrobacter* sp. Phylogenetic analysis confirmed its relation to Enterobacteriaceae members like *Enterobacter, Klebsiella, and Leclercia*. Members of Enterobacteriaceae have been reported as frequent endosymbionts. Campbell et al. [45] studied the phylogeny of symbiotic bacteria of four weevil species (Coleoptera: Curculionidae) and showed that symbionts from taxonomically divergent weevils are mainly members of the *Enterobacteriaceae*. uncultured endosymbiotic bacteria were also detected in this study. DGGE-OTU 4 and 11 were assigned to uncultured bacterial members.

## Conclusions

In conclusion, endosymbiotic bacteria are known to be involved in protecting their host insect against natural antagonists, contributing to insecticide resistance mechanisms, and aiding in food digestion and are, therefore, essential for normal growth and development of their host insect. In this regard, endosymbiotic bacteria could be manipulated, potentially offering new approaches for insect control. Therefore, identification of endosymbiotic bacteria of *O. agamemnon* is an important step in this process. Metagenomic DNA from midguts of *Oryctes* larvae was analyzed for endosymbiotic bacterial communities. Except for the Enterobacteriacaea group, *Oryctes* larvae were found to harbor unique endosymbiotic bacteria when compared with previously reported microbiota. Such distinct microbial community structure and its possible biological function within the insect will provide us with basic information for development of pest control strategies utilizing intrinsic endosymbiotic bacteria. Finally, there is an ultimate question we have to answer, what would be resulted in the absence (either intentional or accidental) of each single symbiont or a specific symbiotic group? If this question is correctly answered, this means a successful control strategy for this insect pest is achieved. Therefore, further studies are now required to clarify the biological function of these endosymbiotic bacteria in *Oryctes* larvae and their potential as novel targets for beetle control.

## Methods

### Insects

*Oryctes agamemnon* larvae were field-collected from a date palm orchard about 80 km north Almadinah Almunawarah region, of Saudi Arabia at longitude (39°11ʹ6ʺ) and latitude (24°47ʹ6ʺ). The 3^rd^ larval instar was dominant in sampling. Samples of larvae were collected in sterilized plastic containers. The larvae were kept in the laboratory for one week prior to dissection to avoid possible infestations from the field and to reduce any potential insecticide residual effects. All stages were kept in plastic containers half-filled with soil and date palm pieces. The larvae were dissected in dissection trays containing 0.65% saline and the midguts were aseptically removed [46]. The midguts were homogenized in a sterile glass homogenizer containing 0.85% saline. The supernatant suspension was used for bacterial enrichment and DNA extraction. Each sample consisted of the content of three pooled midguts taken from three larvae of the same instar. Metagenomic DNA was extracted from soil infested with larvae for comparative purposes.

### DNA extraction and PCR amplification of 16S rRNA genes

Total community DNA was extracted with the Ultra Clean Soil DNA purification kit (Mo Bio Laboratories, Solana Beach, Calif.). Harvested cells were transferred to bead beating tubes and vortexed horizontally for 1 min at room temperature. Supernatant was collected and DNA was precipitated and purified according to the instruction manual. Amplification of 16S rRNA genes for DGGE analysis was performed using GC-clamp primers (EUB341F-GC: 5′-CGCCCGCCGCGCGCGGCGGGCGGGGCGGGGGCACGGGGGGCCTACGGGAGGCAGCAGCAG-3′ and EUB517R: 5′-ATTACCGCGGCTGCTGG-3′) that correspond to positions 341 and 517 in *Escherichia coli* [47]. Amplification were performed in 25 μl reaction vessel containing: 2.5 μl of 10 × *Taq* buffer (100 mM Tris–HCl, pH 8), 1.25 mM MgCl_2_, 100 μM dNTPs (Invitrogen, USA), 1.2 μM forward primer and reverse primer set (Invitrogen, USA), 0.5U *Taq* DNA polymerase (Invitrogen, USA), and about 5 ng of template DNA. PCR was performed in Thermal Cycler (Applied Biosystems 2720, USA). A touchdown PCR program was implemented as follows: initial denaturation step at 95°C for 5 min; 5 cycles of 94°C for 40 sec, annealing at 65°C for 40 sec, and extension at 72°C for 40 sec; 5 cycles of 94°C for 40 sec, annealing at 60°C for 40 sec, and extension at 72°C for 40 sec; 10 cycles of 94°C for 40 sec, annealing at 55°C for 40 sec, and extension at 72°C for 40 sec; 10 cycles of 94°C for 40 sec, annealing at 50°C for 40 sec, and extension at 72°C for 40 sec were performed, followed by a final hold at 72°C for 7 min. Amplicons were analyzed by electrophoresis on 1% agarose gels with the size markers (1 kb DNA ladder, Invitrogen, USA) and visualized using ethidium bromide.

### DGGE

DGGE was performed using Dcode Mutation Detection System (Bio-Rad Laboratories Ltd., Hertfordshire, UK). PCR products were electrophoresed with 0.5 × TAE buffer (1 × TAE buffer is 0.04 M Tris base, 0.02 M sodium acetate, and 10 mM EDTA [pH 7.4]) on 8% acrylamide gel containing 25 to 50% denaturating gradient of formamide and urea. DGGE was conducted at 60°C for 5 h at voltage of 200 V. The gel was stained with SYBR Green I Nucleic acid gel stain (Cambrex Bio Science Rockland, USA), photographed and analyzed for DGGE band profile with a UV gel documentation system (Bio-Rad Laboratories Inc., CA, USA).

### Numerical analysis of the DGGE fingerprints

The DGGE fingerprints were analyzed using Quantity One 1D software (BioRad). The total number of DGGE bands was used to represent OTUs richness [48]. Bacterial diversity was estimated based on densitometric measurements and Shannon diversity index (*H’*) [48,49], Equation (1)

1 $$ H' = - \varSigma {P}_i\left( \ln {P}_i\right) $$

$$ {P}_i={n}_i/{N}_i $$

where *P*_*i*_ is a relative intensity of DNA band in the fingerprint, *n*_*i*_ is densitometrically measured intensity of individual DNA band, and *N*_*i*_ is the total amount of DNA in the fingerprint. The relative intensity of each band (*Pi*) was used to express the relative frequency of each phylotype [50].

### Sequencing of DGGE bands

Dominant DGGE bands were cut off with a sterile scalpel and eluted by incubation in 100 μl of TE buffer at 100°C for 5 min. Supernatant was used as template for PCR amplification. Reamplification of 16Sr RNA genes from excised DNA fragments was performed using bacterial primers EUB314F without GC clamp and EUB517R. Amplification was verified by electrophoresis on 1% agarose gel. PCR products were directly sequenced using a BigDye terminator cycle sequencing [51] at GenoScreen sequencing facility (Genoscreen, Lille, France).

### Sequence analysis

The sequences obtained from the 16S rRNA genes were analyzed by *Genetyx-Win* MFC application software version 4.0. The reference 16S rRNA gene sequences were retrieved from the GenBank database (National Center for Biotechnology Information, National Library of Medicine, USA) [52]. Sequences were compared with their closest matches in GenBank with nucleotide-nucleotide BLAST to obtain the nearest phylogenetic neighbors (www.ncbi.nlm.nih.gov/BLAST/). Sequence alignments were performed by *Clustal W*1.83 XP [53] and phylogenetic trees were constructed using neighbor-joining method [54] using *MEGA6* software [55].

### Accession numbers and data deposition

The 16S rDNA sequences identified in this study have been deposited in the GenBank database under the accession numbers LC009469 to LC009479. The data of the phylogenetic analysis are available from the Dryad Digital Repository: http://dx.doi.org/10.5061/dryad.59h51.

## References

[1] Khalaf MZ, Al Rubeae HF, Al-Taweel A, Naher F (2013). First record of Arabian rhinoceros bettle, *Oryctes agamemnon arabicus* Fairmaire on date palm trees in Iraq. Agric Biol J N Am.

[2] Soltani R, Ikbel C, Hamouda H (2008). Descriptive study of damage caused by the rhinoceros beetle, *Oryctes agamemnon*, and its influence on date palm oases of Rjim Maatoug, Tunisia. J Insect Sci.

[3] Gassouma MS (2004). Pests of the date palm (*Phoenix dactylifera*).

[4] Bedford GO (1980). Biology, ecology and control of palm rhinoceros beetles. Ann Rev Entomol.

[5] Samsudin A, Chew P, Mohd M (1993). *Oryctes rhinoceros*: breeding and damage on oil palms in an oil palm to oil palm replanting situation. Planter.

[6] Ehsine M, Belkadhi M, Chaieb M (2014). Seasonal and nocturnal activities of the rhinoceros borer (Coleoptera: Scarabaeidae) in the north Saharan oases ecosystems. J Insect Sci.

[7] Soltani R (2010). The rhinoceros beetle *Oryctes agamemnon arabicus* in Tunisia: current challenge and future management perspectives. Tunisia J Plant Prot.

[8] Lundgren J, Michael R, Chee-Sanford J (2007). Bacterial communities within digestive tracts of ground beetles (Coleoptera: Carabidae). Ann Entomol Soc Am.

[9] Ferrari J, Vavre F (2011). Bacterial symbionts in insects or the story of communities affecting communities. Phil Trans R Soc B.

[10] Hongoh Y (2010). Diversity and genomes of uncultured microbial symbionts in the termite gut. Biosci Biotechnol Biochem.

[11] Engel P, Moran NA (2013). The gut microbiota of insects - diversity in structure and function. FEMS Microbiol Rev.

[12] Colman DR, Toolson EC, Takacs-Vesbach CD (2012). Do diet and taxonomy influence insect gut bacterial communities?. Mol Ecol.

[13] Duron O, Bouchon D, Boutin S, Bellamy L, Zhou L, Engelstädter J (2008). The diversity of reproductive parasites among arthropods: *Wolbachia* do not walk alone. BMC Biol.

[14] Hilgenboecker K, Hammerstein P, Schlattmann P, Telschow A, Werren JH (2008). How many species are infected with *Wolbachia*? A statistical analysis of current data. FEMS Microbiol Lett.

[15] Zindel R, Gottlieb Y, Aebi A (2011). Arthropod symbioses: a neglected parameter in pest- and disease-control programs. J Appl Ecol.

[16] Oliver KM, Degnan PH, Burke GR, Moran NA (2010). Facultative symbionts in aphids and the horizontal transfer of ecologically important traits. Ann Rev Entomol.

[17] Hirsch J, Strohmeier S, Pfannkuchen P, Reineke A (2012). Assessment of bacterial endosymbiont diversity in *Otiorhynchus* spp. (Coleoptera: Curculionidae) larvae using a multitag 454 pyrosequencing approach. BMC Microbiol.

[18] Moya A, Pereto J, Gil R, Latorre A (2008). Learning how to live together: genomic insights into prokaryote-animal symbioses. Nature Rev Genet.

[19] Moran NA, McCutcheon JP, Nakabachi A (2008). Genomics and evolution of heritable bacterial symbionts. Annu Rev Genet.

[20] Douglas AE (2007). Symbiotic microorganisms: untapped resources for insect pest control. Trends Biotechnol.

[21] Beard CB, Mason PW, Aksoy S, Tesh RB, Richards FF (1992). Transformation of an insect symbiont and expression of a foreign gene in the Chagas disease vector *Rhodnius prolixus*. Amer J Trop Med Hyg.

[22] Avidano L, Gamalero E, Cossa GP, Carraro E (2005). Characterization of soil health in an Italian polluted site by using microorganisms as bio-indicators. Appl Soil Ecol.

[23] Amann R, Ludwig W, Schleifer KH (1995). Phylogenetic identification and in situ detection of individual microbial cells without cultivation. Microbiol Rev.

[24] Head IM, Saunders JR, Pickup RW (1998). Microbial evolution, diversity, and ecology: a decade of ribosomal RNA analysis of uncultivated microorganisms. Microb Ecol.

[25] Hugenholtz P, Goebel B, Pace N (1998). Impact of culture-independent studies on the emerging phylogenetic view of bacterial diversity. J Bacteriol.

[26] Muyzer G (1999). DGGE/TGGE, a method for identifying genes from natural communities. Curr Opin Microbiol.

[27] Muyzer G, Brinkhoff T, Nübel U, Santegoeds C, Schäfer H, Wawer C, Akkermans ADL, van Elsas JD, de Bruijn FJ (1997). Denaturing Gradient Gel Electrophoresis (DGGE) in Microbial Ecology, p. 1–27. Molecular Microbial Ecology Manual.

[28] Marsh TL (1999). Terminal restriction fragment length polymorphism (TRFLP): an emerging method for characterizing diversity among homologous populations of amplification products. Curr Opin Microbiol.

[29] Gelsomino A, Keijzer WA, Cacco G, Van Elsas JD (1999). Assessment of bacterial community structure in soil by polymerase chain reaction and denaturing gradient gel electrophoresis. J Microbiol Meth.

[30] Riemann L, Steward GF, Fandino LB, Campbell L, Landry MR, Azam F (1999). Bacterial community composition during two consecutive NE Monsoon periods in the Arabian Sea studied by denaturing gradient gel electrophoresis (DGGE) of rRNA genes. Deep-Sea Res.

[31] Smalla K, Wieland G, Buchner A, Zock A, Parzy J, Kaiser S (2001). Bulk and rhizosphere soil bacterial communities studied by denaturing gradient gel electrophoresis: plant-dependent enrichment and seasonal shifts revealed. Appl Environ Microbiol.

[32] Ritz K, Mac Nicol JW, Nunan N, Grayston S, Millard P, Atkinson A (2004). Spatial structure in soil chemical and microbiological properties in upland grassland. FEMS Microbiol Ecol.

[33] Sun HY, Deng SP, Raun WR (2004). Bacterial community structure and diversity in a century-old manure-treated agro-ecosystem. Appl Environ Microbiol.

[34] Whiteley AS, Bailey MJ (2000). Bacterial community structure and physiological state within an industrial phenol bioremediation system. Appl Environ Microbiol.

[35] Andert J, Marten A, Brandl R, Brune A (2010). Inter- and intraspecific comparison of the bacterial assemblages in the hindgut of humivorous scarab beetle larvae (*Pachnoda* spp.). FEMS Microbiol Ecol.

[36] Lauzon CR, Sjogren RE, Prokopy RJ (2000). Enzymatic capabilities of bacteria associated with apple maggot flies: a postulated role in attraction. J Chem Ecol.

[37] Adams L, Boopathy R (2005). Isolation and characterization of enteric bacteria from the hindgut of Formosan termite. Biores Technol.

[38] Tagliavia M, Messina E, Manachini B, Cappello S, Quatrini P (2014). The gut microbiota of larvae of *Rhynchophorus ferrugineus* Oliver (Coleoptera: Curculionidae). BMC Microbiol.

[39] Fujiwara A, Maekawa K, Tsuchida T. Genetic groups and endosymbiotic microbiota of the *Bemisia tabaci* species complex in Japanese agricultural sites. J Appl Entomol Online. 2014, doi: 10.1111/jen.12171.

[40] Desai A, Bhamre P (2012). Diversity of gut bacterial fauna of *Oryctes monocerus* linnaeus (coleoptera: scarabaeidae). Bionano Front.

[41] Huang S, Sheng P, Zhang H (2012). Isolation and identification of cellulolytic bacteria from the gut of *Holotrichia parallela* larvae (Coleoptera: Scarabaeidae). Int J Mol Sci.

[42] Sanchez-Contreras M, Vlisidou I (2008). The diversity of insect-bacteria interactions and its applications for disease control. Biotech Gene Eng Rev.

[43] Chandler J, Lang J, Bhatnagar S, Eisen J, Kopp A (2011). Bacterial communities of diverse Drosophila species: ecological context of a host–microbe model system. PLoS Genet.

[44] Ovreas L (2000). Population and community level approaches for analyzing microbial diversity in natural environments. Ecol Lett.

[45] Campbell B, Bragg T, Turner C (1992). Phylogeny of symbiotic bacteria of four weevil species (Coleoptera:Curculionidae) based on analysis of 16s ribosomal DNA. Insect Biochem Molec Biol.

[46] Lemke T, Stingl U, Egert M, Friedrich MW, Brune A (2003). Physicochemical conditions and microbial activities in the highly alkaline gut of the humus-feeding larva of *Pachnoda ephippiata* (Coleoptera: Scarabaeidae). Appl Environ Microbiol.

[47] Muyzer G, De Waal EC, Uitterlinden AG (1993). Profiling of complex microbial populations by denaturing gradient gel electrophoresis analysis of polymerase chain reaction-amplified genes coding for 16S rRNA. Appl Environ Microbiol.

[48] Duarte S, Pascoal C, Garabétian F, Cássio F, Charcosset JY (2009). Microbial decomposer communities are mainly structured by the trophic status in circumneutral and alkaline streams. Appl Enviro Microbiol.

[49] Ping L, Yanxin W, Yanhong W, Kun L, Lei T (2010). Bacterial community structure and diversity during establishment of an anaerobic bioreactor to treat swine wastewater. Water Sci Technol.

[50] Moreirinha C, Duarte S, Pascoal C, Cássio F (2011). Effects of cadmium and phenanthrene mixtures on aquatic fungi and microbially mediated leaf litter decomposition. Arch Environ Cont Toxicol.

[51] Sanger F, Nicklen S, Coulson A (1977). DNA sequencing with chain-terminating inhibitors. Biochemistry.

[52] Altschul SF, Madden TL, Schäffer AA, Zhang J, Zhang Z, Miller W (1997). Gapped blast and psi-blast: a new generation of protein database search programs. Nucleic Acids Res.

[53] Thompson D, Gibson J, Plewinak F, Jeanmougin F, Higgins G (1997). The Clastal X windows interface: flexible strategies for multiple sequence alignment aided by quality analysis tools. Nuc Acid Res.

[54] Saitou N, Nei M (1987). The neighbor-joining method: a new method for reconstructing phylogenetic trees. Mol Biol Evol.

[55] Kumar S, Tamura K, Nei M (2004). MEGA3: an integrated software for molecular evolutionary genetics analysis and sequence alignment. Brief Bioinform.

